# Supplementary material for: Human neutralizing antibodies to cold linear epitopes and subdomain 1 of the SARS-CoV-2 spike glycoprotein
Source: Sci Immunol. 2023 Jan 26:eade0958. doi: 10.1126/sciimmunol.ade0958 (PMC9972897; doi:10.1126/sciimmunol.ade0958)
Supplement: Supplementary file 1 — Figs. S1 to S8 Tables S1 and S2 [file sciimmunol.ade0958_sm.pdf]

Science Immunology
AAAS

Supplementary Materials for

Human neutralizing antibodies to cold linear epitopes and subdomain 1 of the SARS-CoV-2 spike glycoprotein

Filippo Bianchini *et al.*

Corresponding authors: Daniel Ruzek, ruzekd@paru.ca.cz; Luca Varani, luca.varani@irb.usi.ch; Andrea Cavalli, andrea.cavalli@irb.usi.ch; Christopher O. Barnes, cobarnes@stanford.edu; Davide F. Robbiani, drobbiani@irb.usi.ch

Sci. Immunol. **8**, eade0958 (2023)
DOI: 10.1126/sciimmunol.ade0958

The PDF file includes:

Figs. S1 to S8
Tables S1 and S2

Other Supplementary Material for this manuscript includes the following:

Data files S1 to S3
MDAR Reproducibility Checklist

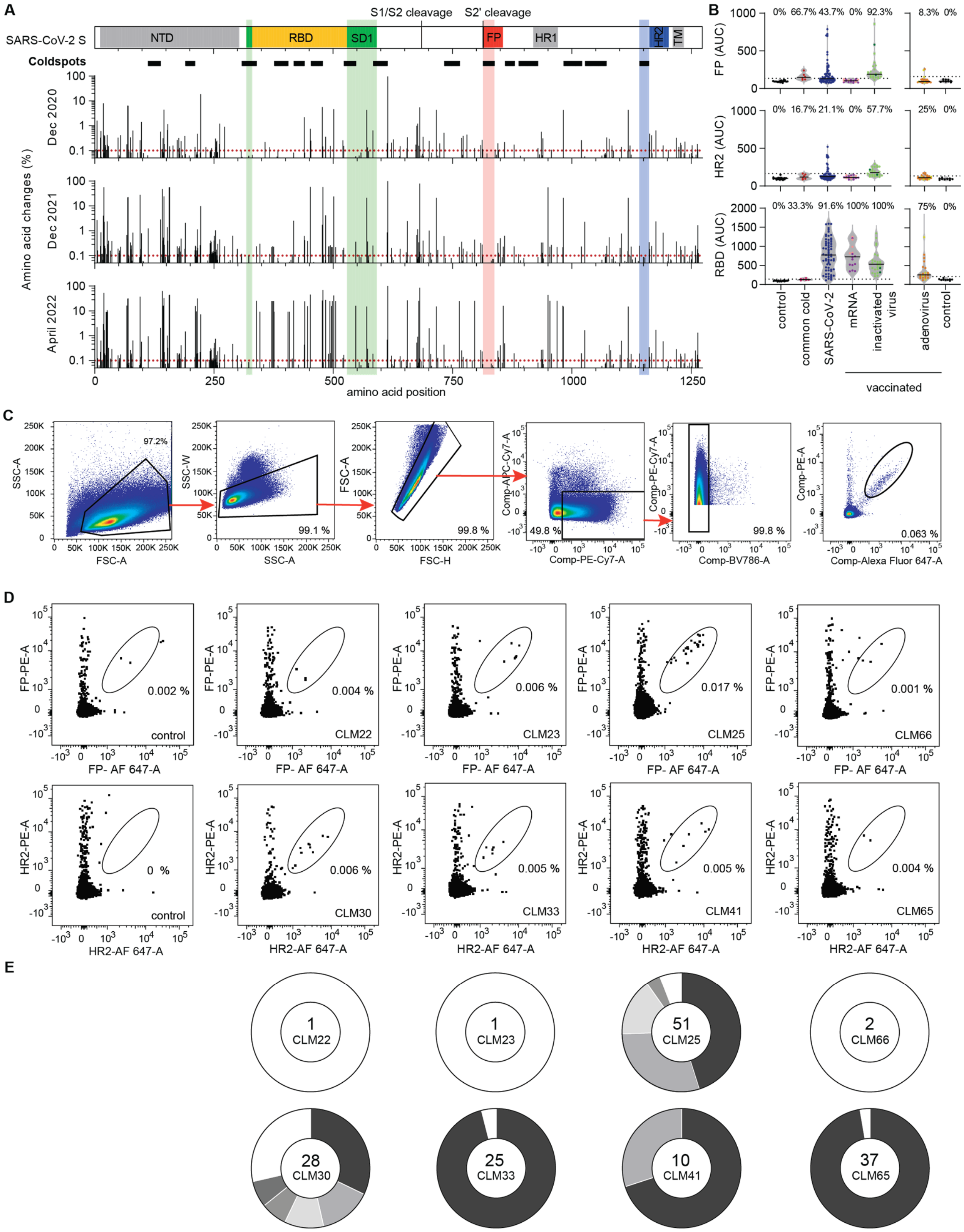

Fig. S1. Identification of coldspot antibodies. (A) Cartoon diagram of the SARS-CoV-2 S with highlighted coldspot regions at the fusion peptide (FP, red) and near heptad repeat 2 (HR2, blue), as well as subdomain 1 (SD1, green). The thick horizontal lines underneath indicate the location of all coldspots, and the histograms show the frequency of aa changes throughout S at different time points: 31.12.2020 (671,006), 31.12.2021 (7,526,116) and 29.04.2022 (10,480,461 sequences from GISAID). **(B)** ELISA reactivity of plasma IgG antibodies to FP (top) and HR2 (middle) peptides or to SARS-CoV-2 RBD (bottom). SARS-CoV-2 (n=67) and common cold CoV (n=6) convalescent samples are compared to COVID-19 vaccinees (mRNA: BNT162b2 n=11, purple; mRNA-1273 n=5, pink;

inactivated virus: Sinovac n=2, dark green; Sinopharm n=24, light green; adenovirus: ChAdOx1-S n=19, orange; Ad26.COV2.S n=4, yellow), and to non-infected controls (n=25). Area Under the Curve (AUC); average of two experiments. The percentage of samples with antibody levels 4 standard deviations above controls is shown on top. **(C)** Gating strategy for sorting peptide-specific or SD1-enriched B cells by flow cytometry. **(D)** Identification of FP-peptide specific (top) and HR2-peptide specific (bottom) B cells by flow cytometry. Numbers indicate percentage of gated double-positive cells. **(E)** Clonal analysis of antibody sequences derived from peptide-specific B cells identified in **(D)**. Pie charts show the total number of antibody sequences (center); the size of the slices is proportional to the number of clonally related sequences. White slices indicate antibody sequences that are not part of a clone.

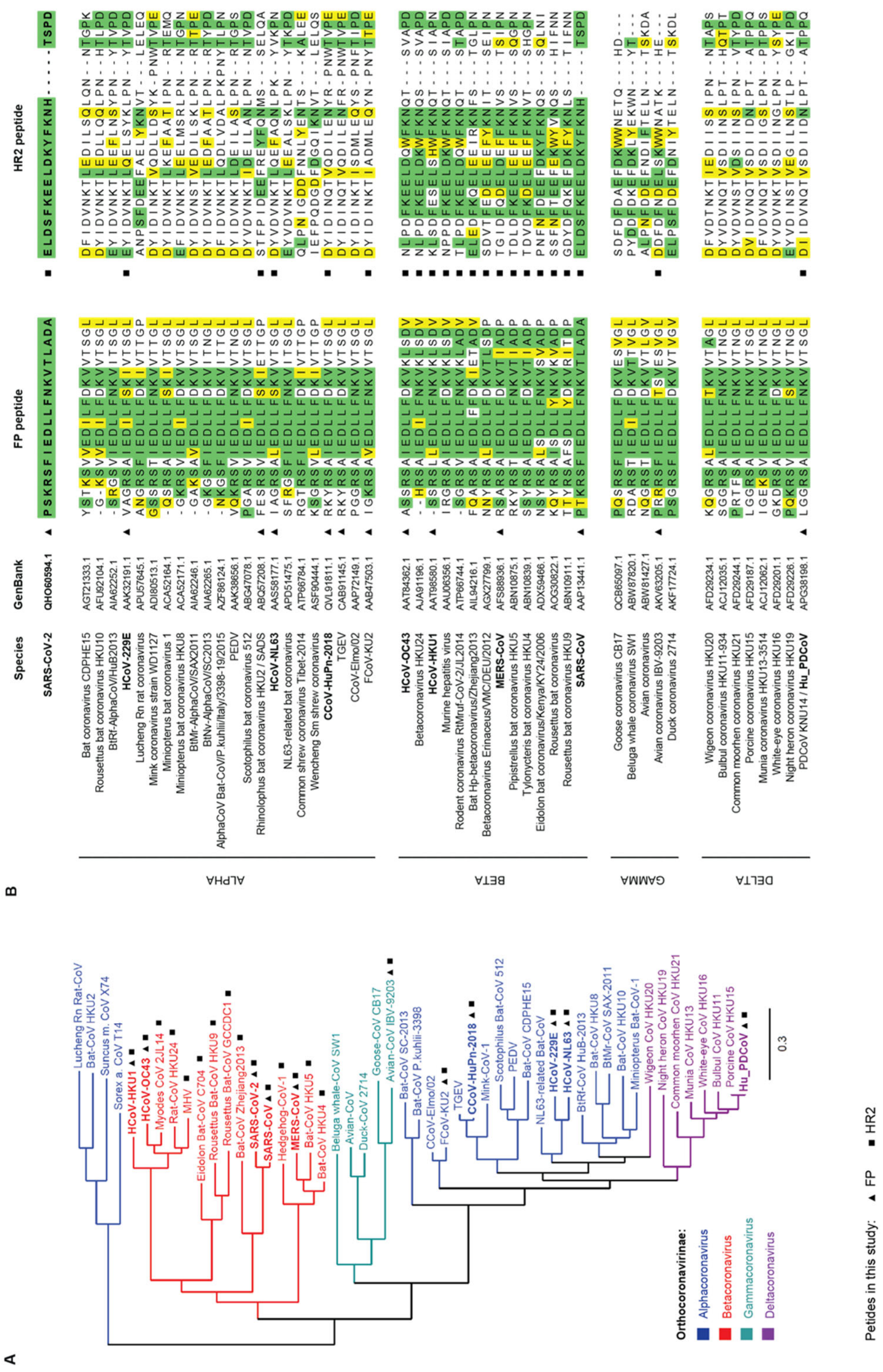

Fig. S2. Similarity of peptides at the FP and HR2 of Orthocoronavirinae. (A) Phylogenetic analysis of CoV species based on the aa sequence of S. **(B)** Alignment of SARS-CoV-2 FP and HR2 peptide sequences with those corresponding to related CoVs. In green identical residues; in yellow residues that are similar by side chain functionality.

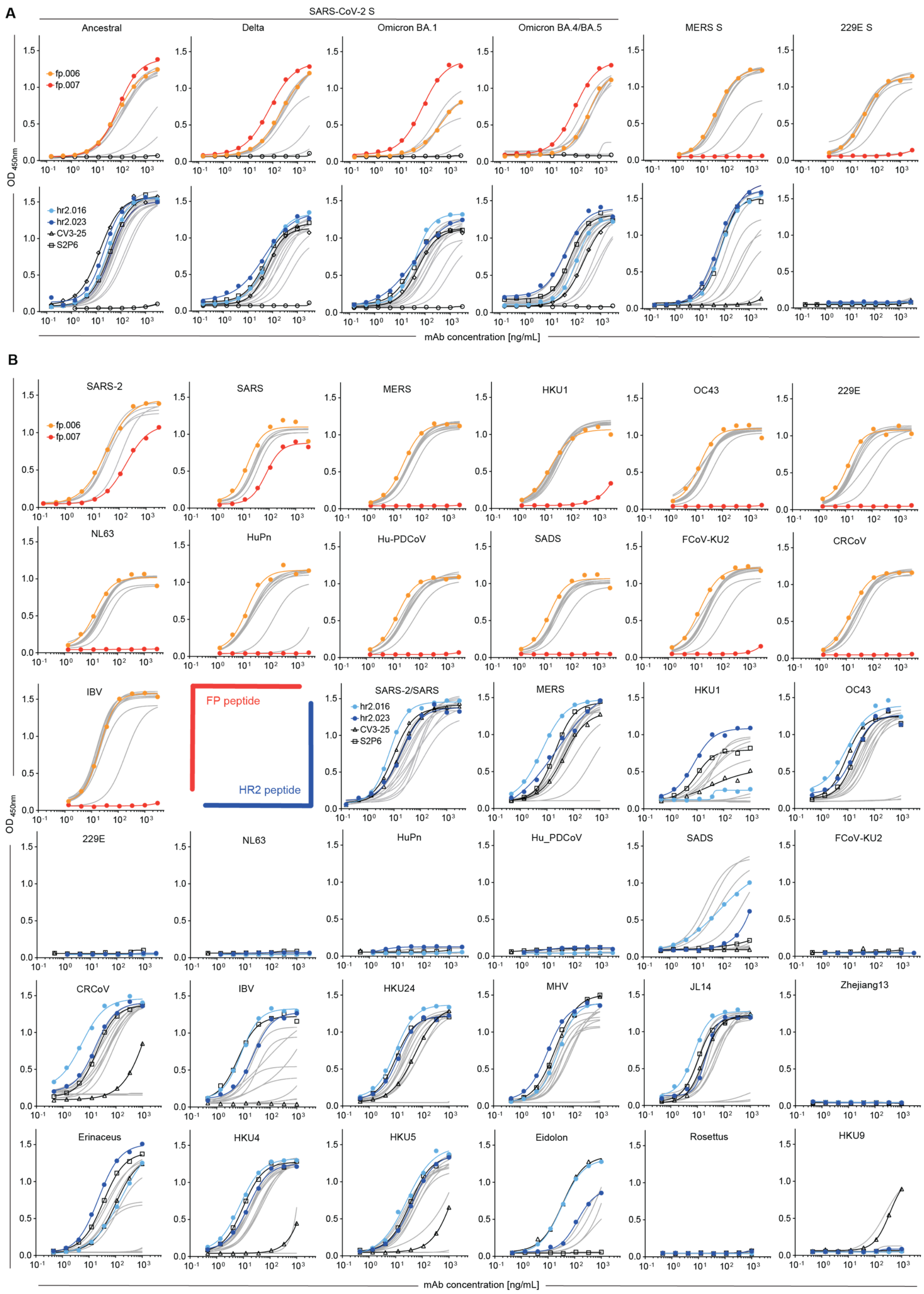

Fig. S3. Broadly crossreactive binding of FP and HR2 monoclonal antibodies. (**A** and **B)** Graphs show ELISAs measuring monoclonal antibodies reactivity to S **(A)** and to coldspot peptides **(B)** of coronaviruses. Mean of two independent experiments.

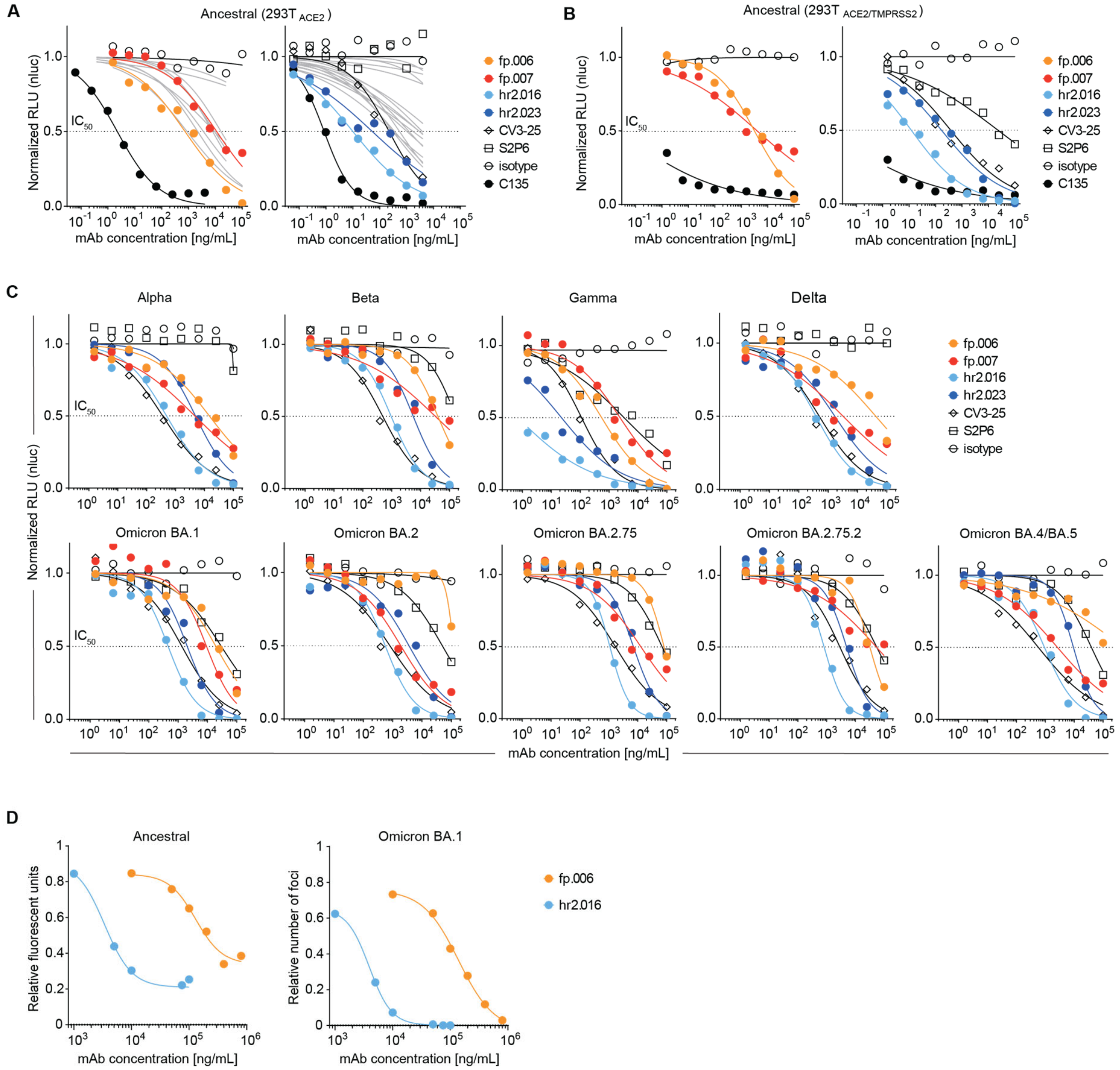

Fig. S4. Broad neutralization by FP and HR2 monoclonal antibodies. (A and B) Neutralization of ancestral SARS-CoV-2 pseudovirus. Graphs show normalized relative luminescence values in cell lysates of $293T_{ACE2}$ **(A)** or $293T_{ACE2/TMPRSS2}$ **(B)** cells 48 hours after infection with nanoluc-expressing SARS-CoV-2 pseudovirus in the presence of increasing concentrations of monoclonal antibodies to the FP (top) and HR2 (bottom). Isotype control and antibodies C135 (4), CV3-25 and S2P6 were assayed alongside for comparison; additional antibodies in grey (data file S2). Mean of at least two independent experiments. **(C)** Same as in **(A)**, but for SARS-CoV-2 pseudoviruses corresponding to VOC. **(D)** Neutralization of SARS-CoV-2 ancestral and Omicron BA.1 by Focus Reduction Neutralization Test (FRNT).

A

	heavy chain														light chain			
	CDRH1	CDRH2							CDRH3					FR1	CDRL3			
Residue Number	31	52	52A	53	55	56	57	58	97	98	99	100A	100F	1	93	94	95	95A
fp.006 Residue	N	S	Y	E	R	N	K	K	F	L	S	Y	Y	E	D	W	P	L
BSA (Å ²)	13	13	83	13	46	69	3	46	18	67	2	77	64	14	12	76	49	21

B

	fusion peptide																	
Residue Number	813	814	815	816	817	818	819	820	821	822	823	824	825	826	827	828	829	830
Conservation (%)	22	14	82	100	18	78	94	100	86	98	96	37	98	86	12	14	18	29
FP Residue	S	K	R	S	F	I	E	D	L	L	F	N	K	V	T	L	A	D
BSA (Å ²)	6	22	172	48	0	57	67*	48	0	89	175	3	71	0	0	22	0	0

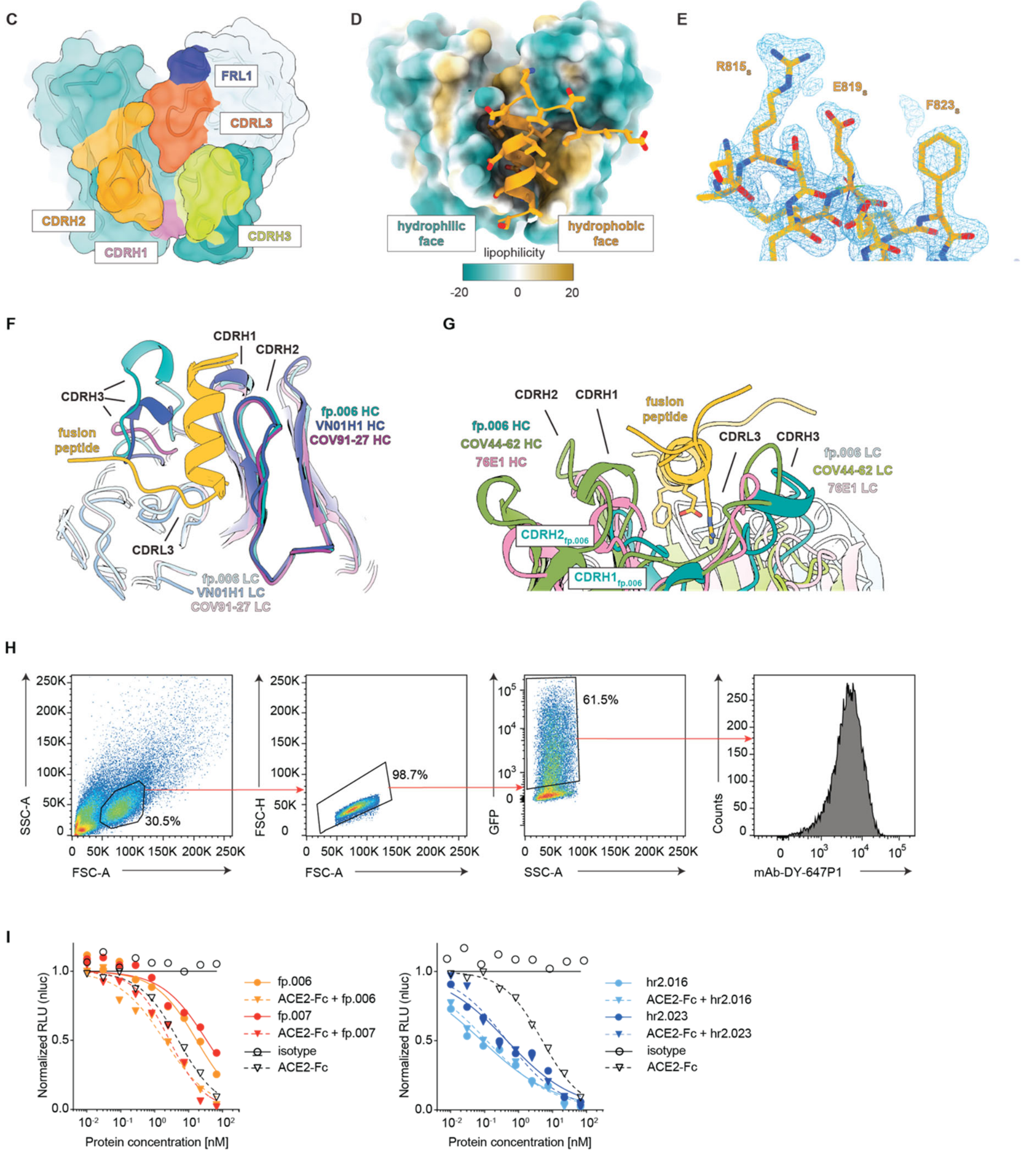

Fig. S5. Structure of fp.006 in complex with peptide antigen and effects of ACE2 engagement. (A) Contributions of fp.006 heavy and light chain paratope residues to the binding of the fusion peptide. Residue number, identity, and buried surface area (BSA) are shown, and colored as in panel **(C)**. **(B)**

Per residue contributions of the FP to the fp.006 epitope. Conservation calculated from the 49 CoV sequences listed in fig. S2B and BSA calculations per FP residue are listed. *Indicates that the BSA calculation does not include contacts mediated by water molecules. **(C)** Surface representation of the fp.006 paratope with interacting residues colored according to their CDR loop identity. Non-interacting heavy chain and light chain residues are colored teal and light teal, respectively. **(D)** Surface representation of the fp.006 paratope with per residue lipophilicity calculations shown. More hydrophobic patches are colored orange and more hydrophilic patches are colored teal, as calculated by ChimeraX. **(E)** Representative electron density for a portion of the FP contoured at 1.7σ. **(F)** Overlay of complex structures between the SARS-CoV-2 fusion peptide and fp.006 (this study; PDB 8D47), VN01H1 (PDB 7SKZ), and COV91-27 (PDB 8D6Z) illustrates a convergent binding mode independent of V gene usage. Structures are aligned on their fusion peptide chains and colored as shown. **(G)** Overlay of complex structures between the SARS-CoV-2 fusion peptide and fp.006 (this study; PDB 8D47), COV44-62 (PDB 8D36), and 76E1 (PDB 7X9E) Fabs, illustrating that anti-FP mAbs of different poses bind to the same face of the amphipathic fusion peptide α-helix, which includes highly-conserved residues, which are shown as sticks. **(H)** Gating strategy for measuring the binding of monoclonal antibodies to the SARS-CoV-2 S expressed by 293T cells by flow cytometry. **(I)** Normalized relative luminescence values in cell lysates of $293T_{ACE2}$ cells after infection with ancestral SARS-CoV-2 pseudovirus in the presence of increasing concentrations of FP or HR2 antibodies alone or as a cocktail with soluble ACE2. Isotype control in black. For the combination of fp.006+ACE2, P=0.0017 over fp.006 and P=0.0047 over ACE2; for the combination of fp.007+ACE2, P=0.0072 over fp.007 and P=0.2 over ACE2 (n=4; Welch's t-test, two-tailed).

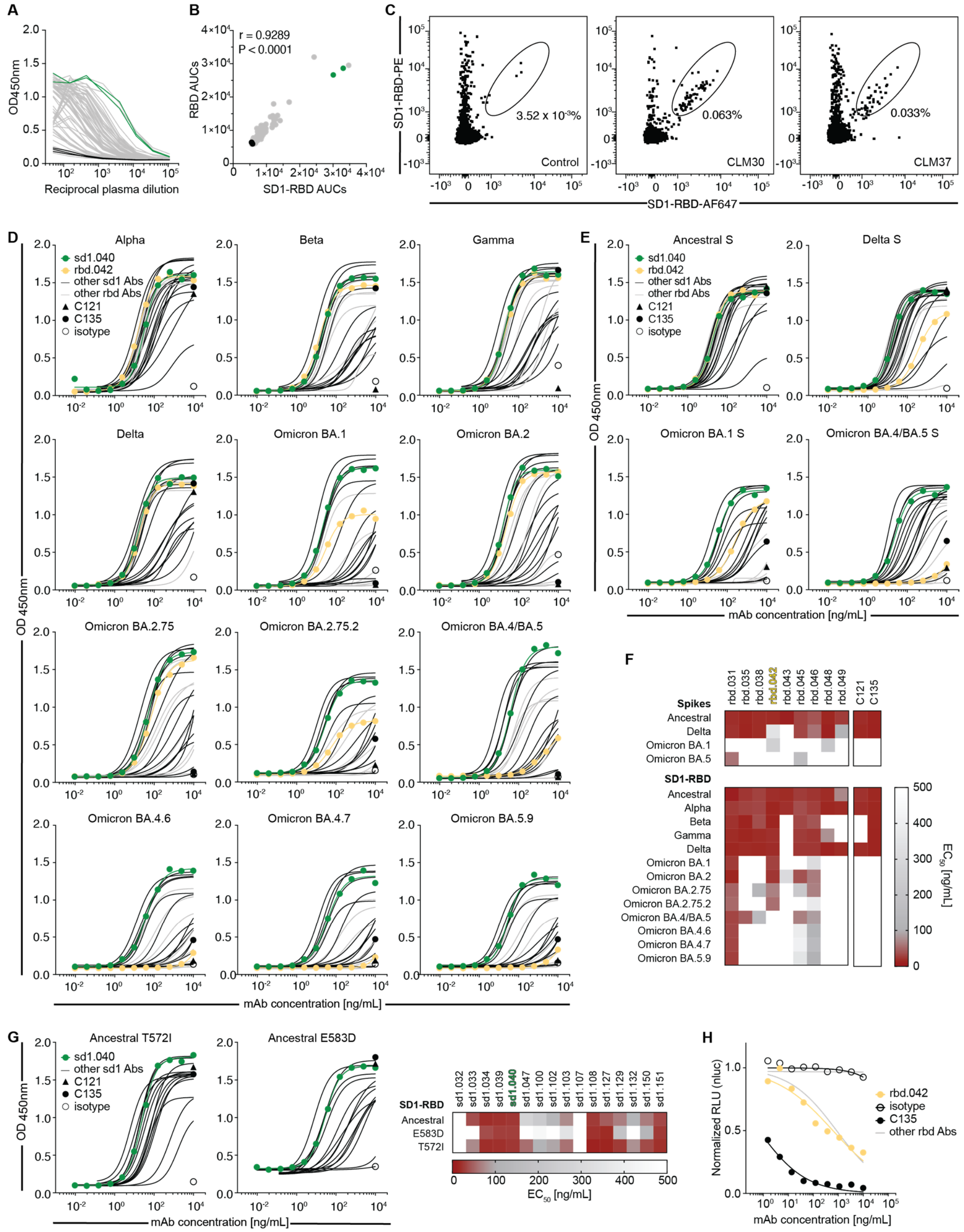

Fig. S6. Identification of SD1 and RBD antibodies. (A) Graph shows ELISAs measuring plasma IgG reactivity to RBD. Negative controls in black. Mean of two independent experiments. **(B)** Correlation of plasma IgG reactivities to SD1-RBD and RBD. Pearson correlation. **(C)** Identification of SD1-enriched B cells by flow cytometry. **(D** and **E)** Graphs show ELISAs measuring monoclonal antibodies reactivity to SD1-RBD **(D)** and S **(E)** corresponding to SARS-CoV-2 VOC. Mean of two independent experiments. **(F)** Heatmaps with the binding (EC_{50}) of RBD monoclonal antibodies to S (top) or SD1-RBD (bottom) proteins corresponding to SARS-CoV-2 VOC. Two experiments. **(G)** Graphs and summary heatmap for SD1 antibodies binding to SD1-RBD mutant proteins T572I and E583D. **(H)** Neutralization of ancestral SARS-CoV-2 pseudovirus by antibodies to the RBD that are broadly crossreactive to VOC.

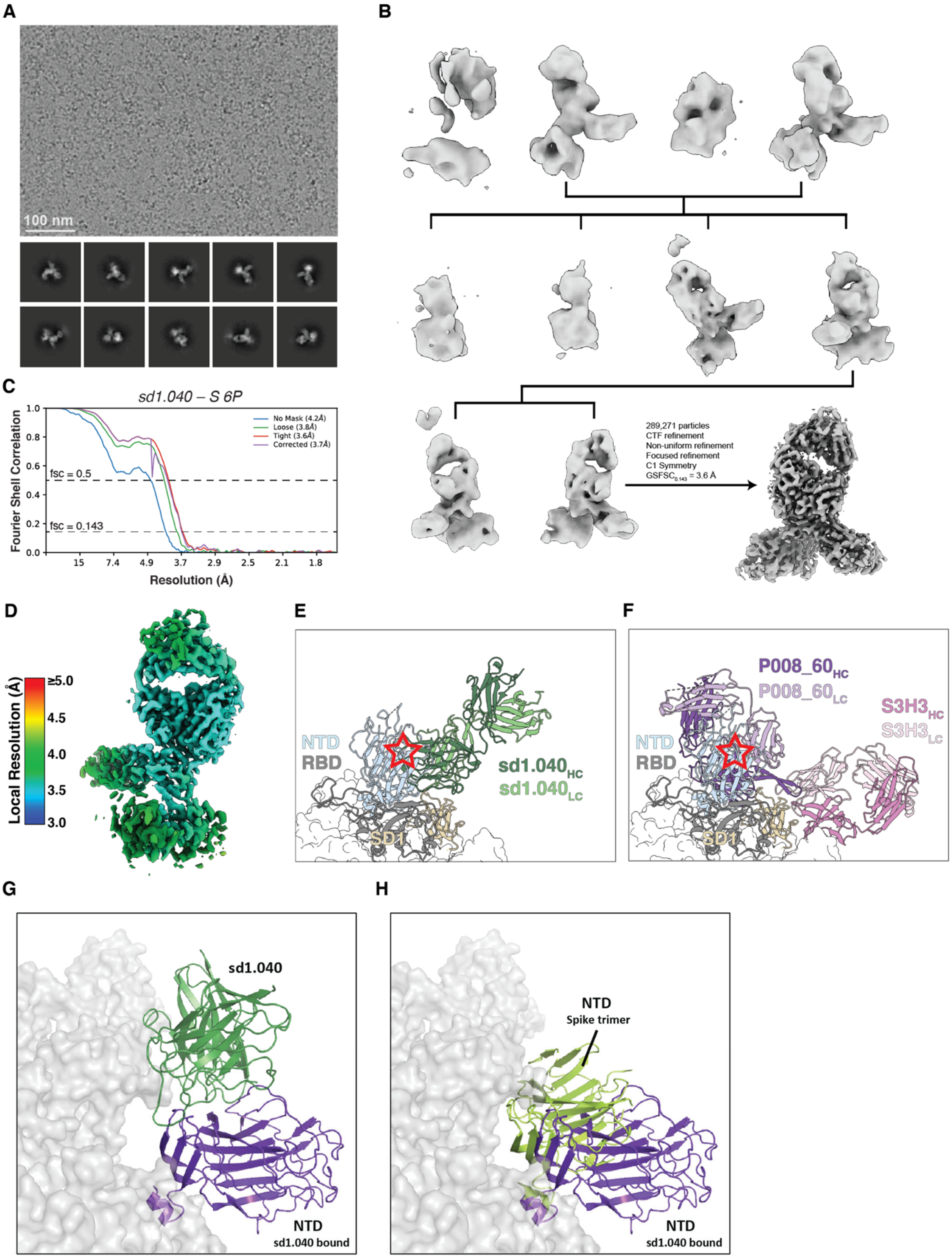

Fig. S7. Structure of sd1.040 in complex with S. (A) Representative micrograph and 2D class averages selected from the sd1.040-S dataset. **(B)** 3D classification workflow and final refinement strategy. **(C)** Gold-standard FSC plot for the final cryo-EM density map. **(D)** Local resolution estimates calculated

in cryoSPARC v3.1. **(E)** Close-up view of the sd1.040 Fab – S complex superimposed onto a prefusion S trimer (PDB 6VXX). Potential clashes with the NTD (cyan) on the neighboring S1 protomer are denoted by a red star. **(F)** Close-up views of the P008_60 Fab (PDB 7ZBU) and S3H3 Fab (PDB 7WD9) superposed onto a prefusion trimeric Spike. Antibody P008_60 shows prominent clashes with the adjacent NTD (red star), while the binding orientation of S3H3 does not clash with neighboring protomers. **(G and H)** According to docking simulations, sd1.040 binds also to the NTD of the adjacent protomer. In **(G)**, NTD moves from the position observed in the experimental structure of the free S to make room for antibody binding (sd1.040 in dark green bound to NTD in purple). In **(H)**, comparison between NTD bound to sd1.040 in simulations (purple) and NTD in the context of free spike trimer (lime).

A

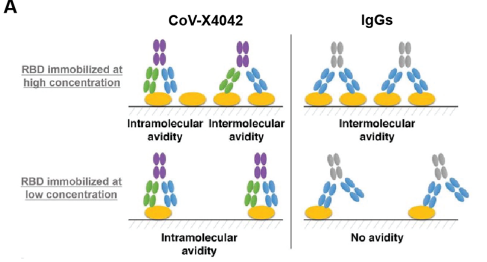

	Immobilized on the chip			
	SD1-RBD 150 nM	SD1-RBD 75 nM	SD1-RBD 15 nM	
**rbd.042 Fab**	1.00	1.01	0.99	**Ka normalized** Ka(Xi nM)/Ka(150 nM)
**rbd.042 IgG**	1.00	0.80	0.62	
**CoV-X4042**	1.00	0.71	0.44	
**rbd.042 Fab**	1.00	1.15	0.80	**Kd normalized** Kd(Xi nM)/Kd(150 nM)
**rbd.042 IgG**	1.00	1.62	2.67	
**CoV-X4042**	1.00	1.17	1.37	

B

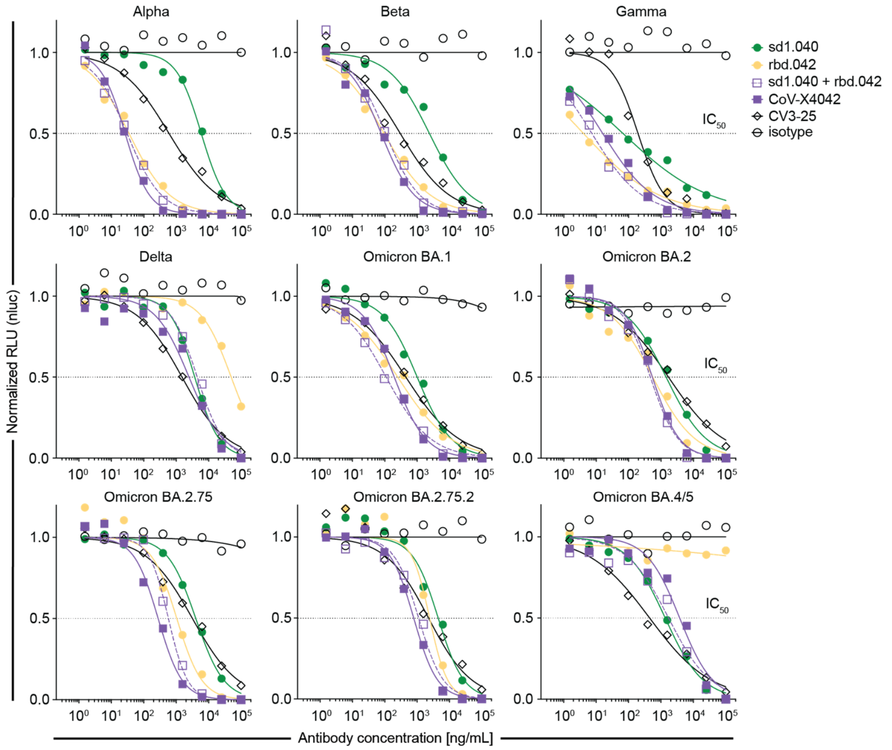

Fig. S8. Binding and neutralizing properties of CoV-X4042. **(A)** Both arms of CoV-X4042 bind simultaneously to the same SD1-RBD molecule. Left, diagram showing that at high SD1-RBD concentrations, monoclonal antibodies have avidity effects owing to intermolecular binding (which results in a slower kd), but this is not the case at low SD1-RBD concentrations, because bivalent binding to a single SD1-RBD is impossible. By contrast, the bispecific antibody has avidity at both high and low concentrations, since bivalent binding to its two epitopes on a single SD1-RBD is possible. ka is not affected by avidity. Right, table summarizing the SPR results used to determine ka and kd of IgG, Fab and the bispecific antibody at different concentrations of immobilized SD1-RBD, which confirm experimentally that CoV-X4042 engages bivalently on a single SD1-RBD. **(B)** Neutralization of VOC by CoV-X4042 and parental monoclonal antibodies.

Table S1. Crystallographic data collection and refinement statistics

	**fp.006-fusion peptide**
**PDB ID**	8D47
**Data collection**[a,b]	
Space group	P 1 2_1 1
Cell dimensions	
a, b, c (Å)	90.0, 73.1, 90.1
α, β, γ (°)	90.0, 109.0, 90.0
Resolution (Å)	37.9 - 2.0 (2.04 - 2.00)
R_{merge}	0.117 (0.996)
I / σI	6.1 (1.5)
$CC_{1/2}$ (%)	0.988 (0.372)
Completeness (%)	97.9 (98.8)
Redundancy	3.0 (3.0)
**Refinement**	
Resolution (Å)	37.9 - 2.0
No. reflections	73148 (4545)
R_{work} / R_{free}	.1836/.2378
No. atoms	7723
Protein	6926
Ligand/ion	45
Water	752
B-factors (mean)	
Protein	30.7
Ligand/ion	41.7
Water	36.5
R.m.s. deviations	
Bond lengths (Å)	0.007
Bond angles (°)	0.840

[a]Data were derived from a single crystal
[b]Values in paratheses describe the highest resolution shell

Table S2. Cryo-EM data collection, refinement and validation statistics

	**sd1.040 – S 2P**<br>(EMDB-27177)<br>(PDB 8D48)
**Data collection and processing**	
Magnification	105,000x
Voltage (kV)	300
Electron exposure (e–/Å^2)	60
Defocus range (μm)	1.0 - 2.5
Pixel size (Å)	0.8521
Symmetry imposed	C1
Initial particle images (no.)	9,894
Final particle images (no.)	9,469
Map resolution (Å)	
FSC threshold (0.143)	3.7
Map resolution range (Å)	3.7 – 4.2
**Refinement**	
Initial model used (PDB code)	6VXX, 5AVE, 7D0D
Model resolution (Å)	
FSC threshold (0.143)	3.6
Model resolution range (Å)	3.5 – 3.8
Map sharpening B factor (Å^2)	114
Model composition	
Non-hydrogen atoms	5,545
Protein residues	714
Ligands	2
B factors (Å^2)	
Protein	44
Ligand	132
R.m.s. deviations	
Bond lengths (Å)	0.004
Bond angles (°)	0.8
Validation	
MolProbity score	2.0
Clashscore	11.7
Poor rotamers (%)	0
Ramachandran plot	
Favored (%)	92.8
Allowed (%)	7.2
Disallowed (%)	0
